# The Effect of Heat Stress on Broiler Meat Quality and the Mechanisms Underlying Muscle Acidification: An In Vivo and In Vitro Study

**DOI:** 10.3390/metabo16050298

**Published:** 2026-04-27

**Authors:** Yongjie Xu, Zhuoxian Weng, Xunhe Huang, Xiaohuan Chao, Xiquan Zhang, Xiaonan Zhang, Qingbin Luo

**Affiliations:** 1 Guangdong Provincial Key Laboratory of Conservation and Precision Utilization of Characteristic Agricultural Resources in Mountainous Areas, Jiaying University, Meizhou 514000, China; 2 Guangdong Key Laboratory of Agricultural Animal Genomics and Molecular Breeding, Key Laboratory of Chicken Genetic Breeding and Reproduction, Ministry of Agriculture, South China Agricultural University, Guangzhou 510642, China

**Keywords:** heat stress, yellow-feathered broilers, flavor substance, PSE meat, post-slaughter refrigeration, glycometabolism

## Abstract

**Background:** This study investigated how chronic heat stress affects meat quality and post-slaughter muscle acidification in slow-growing yellow-feathered broilers, focusing on the roles of ALDOB and HSP90B1 in glycometabolism. **Methods:** From 100 to 120 days of age, broilers were kept either under thermoneutral conditions (25 ± 1 °C, N group) or cyclic heat stress (32 ± 1 °C for 9 h/day, H group). Meat quality traits (pH, shear force, drip loss, color) were measured at 0, 24, and 48 h of refrigeration (4 °C). Free amino acid and fatty acid profiles were analyzed. DF-1 cells were exposed to 43 °C for functional assays of *ALDOB* and *HSP90B1*. **Results:** Chronic heat stress reduced body weight, altered flavor precursors, and induced PSE-like characteristics (lower pH, higher shear force, increased drip loss, paler color), especially in leg muscles. *ALDOB* and *HSP90B1* were upregulated in both tissues and cells. *ALDOB* overexpression promoted glucose consumption, while *HSP90B1* suppressed lactic acid production. **Conclusions:** Chronic heat stress impairs growth and flavor precursors and exacerbates post-slaughter muscle acidification (primarily driven by ATP hydrolysis, with lactic acid as a secondary contributor). *ALDOB* and *HSP90B1* may dually regulate glycometabolism under heat stress.

## 1. Introduction

Heat stress is one of the most significant environmental challenges facing the global poultry industry. With rising global temperatures and increasing frequency of extreme weather events, the detrimental effects of heat stress on broiler production have become more pronounced [1]. Unlike mammals, poultry lack functional sweat glands and rely primarily on accelerated respiration to dissipate body heat. The optimal growth temperature for broilers is 18–21 °C; when ambient temperature exceeds 25 °C, feed intake progressively declines while water consumption increases [2]. At temperatures approaching 30 °C, birds essentially cease feeding, exhibit open-mouth panting, and may ultimately succumb to heat exhaustion [2]. These thermoregulatory limitations make broilers particularly susceptible to heat stress, especially under intensive farming conditions.

Chronic heat stress has been documented to suppress feed intake in broilers (Gallus gallus domesticus), reduce growth rate, impair immune function, and compromise meat quality [1,3,4]. A key consequence of heat stress is the increased incidence of pale, soft, and exudative (PSE) meat-a quality defect characterized by light color, soft texture, and reduced water-holding capacity (WHC) [3,4]. PSE meat arises from rapid postmortem ATP hydrolysis and the accompanying glycolysis, which together accelerate pH decline while the carcass temperature remains elevated; the combination of low pH and high temperature causes partial denaturation of muscle proteins, leading to the characteristic pale color and poor WHC [5]. In normal broiler meat, postmortem pH typically declines gradually from approximately 6.2–6.5 immediately after slaughter to an ultimate pH of 5.7–5.9 within 24 h [6,7]. In PSE-affected meat, however, pH can drop below 5.8 within the first hour postmortem while muscle temperature remains high, resulting in compromised functional properties [5,8]. Classification of PSE meat commonly employs pH thresholds (<5.7–5.9) and lightness (L*) values (>49–62), depending on the specific criteria used [3,6,9]. These changes not only diminish consumer acceptability but also cause substantial economic losses due to reduced processing yields and increased purge in packaged products [5].

Meat quality is a multifactorial attribute encompassing pH, shear force (tenderness), WHC, meat color (L*, a*, b*), and flavor [10]. Among these, WHC is particularly critical for processing characteristics and consumer perception, as reduced WHC leads to increased drip loss during storage and cooking, compromising both yield and juiciness [11]. The rate and extent of postmortem glycolysis are key determinants of ultimate meat quality [12]. High temperatures promote PSE meat development through post-slaughter lipid peroxidation, protein denaturation, and alterations in meat color and WHC [13]. Flavor substances—primarily free amino acids and fatty acids—serve as sensory stimuli that collectively determine the characteristic aroma and taste of meat [14]. Prior studies have shown that chronic heat stress increases shear force in broiler breast muscle (with greater shear force indicating elevated toughness) while simultaneously reducing pH, adversely affecting palatability [15]. Heat stress has also been reported to induce inflammation and histological lesions in broiler muscle tissue [16]. During heat stress, the acid-base homeostasis of broilers is disrupted, triggering large-scale organic acid secretion and consequent blood pH depression. Postmortem muscle pH decline is a well-established mechanism resulting primarily from the accumulation of H^+^ ions following ATP hydrolysis, with lactic acid from anaerobic glycolysis being one of several contributing factors [17]. The rate and extent of pH decline are influenced by multiple factors, including the buffering capacity of muscle proteins, the rate of ATP turnover, and the production of metabolic acids. Muscle pH is a critical determinant of meat quality, and understanding its regulation requires consideration of the integrated glycometabolic response rather than focusing solely on lactic acid accumulation.

Despite substantial progress in understanding the physiological effects of heat stress on broiler meat quality, significant research gaps remain. First, although the association between heat stress and PSE meat incidence has been documented, the precise molecular mechanisms linking chronic heat exposure to post-slaughter muscle acidification—particularly the coordinated regulation of glycolytic and gluconeogenic pathways—remain incompletely understood. Second, while aldolase B (*ALDOB*) and heat shock protein 90 beta family member 1 (*HSP90B1*) have been individually implicated in stress responses and metabolic regulation, their functional roles in glycometabolic reprogramming under heat stress conditions have not been systematically characterized, nor has their potential interaction in modulating lactic acid production been explored. Third, previous studies have largely focused on breast meat quality, with limited comparative investigation of leg muscle responses to chronic heat stress during post-slaughter cold storage. These knowledge gaps constrain the development of targeted strategies to mitigate heat-stress-induced deterioration in meat quality.

Aldolase B (*ALDOB*) is an enzyme that catalyzes the reversible cleavage of D-fructose-1,6-bisphosphate to D-glyceraldehyde 3-phosphate and dihydroxyacetone phosphate, participating in both glycolysis and gluconeogenesis [18]. Aberrant *ALDOB* expression has been linked to hereditary fructose intolerance [19], altered aldolase activity, and, consequently, impaired glycometabolism and growth [20]. Notably, whole-genome expression profiling of heat-stressed broilers revealed upregulation of *ALDOB* in leg muscle tissue [21], suggesting a potential role in heat-stress-induced metabolic alterations that may affect meat quality. However, whether *ALDOB* directly contributes to the enhanced glycolytic flux and lactic acid accumulation (one of several contributors to pH decline) observed in heat-stressed muscles remains to be functionally validated.

Heat shock protein 90 kDa beta member 1 (*HSP90B1*, also known as glucose-regulated protein 94, GRP94) is an endoplasmic reticulum (ER)-resident member of the HSP90 family [22]. *HSP90B1* performs unique chaperone functions in the ER and is upregulated during unfolded protein response (UPR) [23]. High *HSP90B1* expression protects muscle cells from intracellular Ca^2+^-mediated damage, whereas reduced levels impair myocyte fusion capacity and induce sarcoplasmic reticulum stress [10]. Such stress elevates intra-reticulum Ca^2+^ concentration, promotes irreversible actin-myosin binding, and may contribute to muscle rigidity and pH decline. Importantly, *HSP90B1* expression in broilers has been significantly correlated with sensory meat quality indicators and blood biochemical indices [10]. Although *HSP90B1* is well recognized for its roles in protein folding and stress adaptation, its involvement in glycometabolic regulation, particularly its potential effects on lactic acid production in heat-stressed muscle, has not been previously investigated.

Against this background, the present study was designed to address the following research questions: (1) What are the effects of chronic cyclic heat stress on the growth performance, flavor substance composition, and post-slaughter meat quality (including pH, shear force, meat color, and PSE-related parameters) of slow-growing yellow-feathered broilers? (2) How do *ALDOB* and *HSP90B1* regulate glycometabolism and lactic acid production under heat stress conditions at the cellular level? (3) We hypothesized that chronic heat stress would negatively impact growth performance and flavor substance deposition, promote PSE-like meat formation and muscle acidification during post-slaughter cold storage, and that these effects would be mediated, at least in part, by the dual regulation of glycometabolism through ALDOB (promoting glucose consumption) and HSP90B1 (suppressing lactic acid production). While postmortem pH decline is primarily driven by ATP hydrolysis, the modulation of lactic acid production represents a secondary but potentially significant pathway under heat stress, warranting investigation. To test these hypotheses, we compared the growth performance, slaughter traits, free amino acid and fatty acid profiles, and meat quality parameters (pH, shear force, meat color at 0, 24, and 48 h post-slaughter) between heat-stressed and non-heat-stressed yellow-feathered broilers. Additionally, we performed cellular overexpression and knockdown experiments in DF-1 cells to elucidate the functional roles of *ALDOB* and *HSP90B1* in glucose consumption and lactic acid production under heat stress. With ongoing restrictions on live poultry trade and the growing promotion of chilled chicken in China [24], understanding the dynamics of meat quality changes during cold storage has become increasingly relevant. The present study, therefore, provides both descriptive characterization of heat stress-induced meat quality deterioration and mechanistic insights into the underlying glycometabolic regulation.

## 2. Materials and Methods

### 2.1. Ethical Statement

All animal procedures were conducted in accordance with the 3R principles (Replacement, Reduction, Refinement) and were approved by the Institutional Animal Care and Use Committee of Jiaying University (Approval No. JYYXLL2025-13). The study was carried out under an experimental animal use license. All birds were humanely euthanized at the end of the experiment.

### 2.2. Animals and Experimental Design

A total of 60 slow-growing yellow-feathered broilers (100 days old, mixed sex, supplied by Guangzhou Jiangfeng Industrial Co., Ltd. (Guangzhou, China)) were randomly allocated into two groups of equal body weight (mean body weight ± SE: 1250 ± 35 g): a non-heat stress group (N group) and a heat stress group (H group), with 30 birds per group. Each group was housed in a separate artificial climate chamber (Conviron, PGV36: 260 cm × 241 cm × 341 cm; Conviron, Winnipeg, MB, Canada), each containing six cages (40 cm × 100 cm × 80 cm, with 5 birds per cage). Thus, the experimental unit was the cage (*n* = 6 cages per treatment, with 5 birds per cage as subsamples). The N group was maintained at a constant temperature of 25 ± 1 °C. The H group was subjected to cyclic heat stress at 32 ± 1 °C from 09:00 to 18:00 daily, and returned to 25 ± 1 °C from 18:00 to 09:00 of the next day. The temperature–humidity index (THI) was calculated as THI = 0.85 × Tdb + 0.15 × Twb (where Tdb = dry bulb temperature, Twb = wet bulb temperature). In the H group during the 32 °C period, the relative humidity was maintained at 75%, resulting in a THI of approximately 28.5, which is considered severe heat stress for broilers. Both groups were exposed to a 16 h light/8 h dark photoperiod (05:00–21:00), with a relative humidity of 75%, and were provided ad libitum access to feed and water. Manure was removed daily. The experiment lasted from 100 to 120 days of age. Body weight was recorded at 110 d and 120 d. To minimize pre-slaughter stress, birds were not fasted prior to slaughter; transport was eliminated by performing slaughter on-site at the experimental facility. All birds were slaughtered within 30 min of removal from the housing chambers.

### 2.3. Sample Collection and Slaughter Performance

At 120 d, all 30 birds per group were slaughtered by cervical bleeding in a randomized order. Before slaughter, approximately 2 mL of peripheral blood was collected from each bird via the brachial (wing) vein, transferred into a heparin sodium anticoagulant tube, and centrifuged at 4 °C and 3000× *g* for 15 min using a refrigerated centrifuge (Eppendorf 5810R, Eppendorf AG, Hamburg, Germany). The plasma was separated, aliquoted, and stored at −80 °C until analysis. Plasma corticosterone concentration was measured using a chicken-specific corticosterone enzyme-linked immunosorbent assay (ELISA) kit (Cayman Chemical Company, Ann Arbor, MI, USA). Slaughter was performed by a single trained operator to ensure consistency. Birds were scalded at 60 °C for 150 s, manually defeathered, and eviscerated. Carcass weight, semi-eviscerated weight, and full-eviscerated weight were recorded. The complete right breast and leg muscles were carefully dissected, and abdominal fat, wings, heart, liver, gizzard (contents and cuticle removed), and proventriculus were separated and weighed. Breast and leg muscle samples were immediately placed at 4 °C in unpackaged condition (i.e., placed on clean stainless steel trays without wrapping) for subsequent meat quality assessments at 0, 24, and 48 h post-slaughter. The cooling rate was monitored using a thermocouple inserted into the geometric center of the breast muscle; the temperature decreased from 38 °C to 4 °C within 90 min post-slaughter.

### 2.4. Meat Quality Traits

Meat quality measurements were performed on 6 birds per group (randomly selected from the 30 birds) at each refrigeration time point (0, 24, and 48 h). All measurements were taken on unpackaged samples stored at 0–4 °C. The pH meter (MIK-PH6.0) was calibrated before each measurement session using standard buffer solutions of pH 4.0, 7.0, and 10.0. The electrode tip was fully inserted into the muscle tissue (approximately 1 cm depth), and three measurements were taken at different locations (cranial, middle, caudal) per sample. Shear force was determined using a texture analyzer (HY-0230) on cooked samples. For cooking, breast and leg muscle samples (1.5 cm thickness) were vacuum-sealed in plastic bags and cooked in a water bath at 80 °C until the internal temperature of 75 °C was reached, then cooled to room temperature. Shear force was measured perpendicular to the muscle fiber direction, and three measurements were taken per sample. Meat color (L*, a*, b* values) was measured with a portable colorimeter (PH-STAR) after a 30 min blooming period at 4 °C under aerobic conditions. The colorimeter was calibrated against a white standard plate (*L* = 93.5, a* = 0.3, b* = 0.4) using illuminant D65 and a 10° observer angle. Three measurements were taken per sample. Water-holding capacity (WHC) was assessed by the filter paper press method: a 2 g muscle sample was placed between two filter papers under a 5 kg weight for 5 min, and the ratio of pressed area to total area was calculated. WHC was expressed as % free water.

### 2.5. Flavor Substance Analysis

Fresh breast and leg muscle samples from six birds per group (randomly selected from the 30 birds) were used for flavor substance analysis. Samples were homogenized, dried to constant weight at 60 °C, and then subjected to free amino acid and fatty acid profiling. Free amino acids were determined by high-performance liquid chromatography (HPLC) using an Agilent 1260 Infinity system (Agilent Technologies, Santa Clara, CA, USA) with a Zorbax Eclipse AAA column (4.6 × 150 mm, 3.5 µm; Agilent Technologies, Santa Clara, CA, USA). Mobile phases were 40 mM Na_2_HPO_4_ (pH 7.8) and acetonitrile:methanol:water (45:45:10, *v*/*v*/*v*) at a flow rate of 1.0 mL/min. Detection was at 338 nm (for primary amino acids) and 262 nm (for secondary amino acids). Fatty acid composition was analyzed by gas chromatography (GC) using an Agilent 7890B system (Agilent Technologies, Santa Clara, CA, USA) equipped with a flame ionization detector and an HP-88 capillary column (100 m × 0.25 mm × 0.2 µm; Agilent Technologies, Santa Clara, CA, USA). The oven temperature was programmed from 100 °C (held 5 min) to 240 °C at 4 °C/min (held 20 min). Helium was the carrier gas at 1.0 mL/min. Lipid extraction followed the Folch method, and methylesterification was performed according to GB 5009.168-2016 [25]. No sensory analysis was performed, as the study focused on instrumental flavor-precursor measurements.

### 2.6. Cell Experiments

Chicken embryo fibroblast cells (DF-1; Shanghai Zhiyan Biotechnology Co., Ltd., Shanghai, China) were used for all cellular assays. Cells were cultured under standard conditions (37 °C, 5% CO_2_) in DMEM (Gibco, Grand Island, NY, USA) supplemented with 10% fetal bovine serum (FBS; Gibco, Grand Island, NY, USA) and 1% penicillin–streptomycin (Gibco, Grand Island, NY, USA). Upon reaching 80–90% confluence, cells were subjected to heat stress at 43 °C for 0, 1, 3, or 6 h. The CO_2_ incubator (Thermo Fisher Scientific, Waltham, MA, USA) was pre-equilibrated at the target temperature for 2 h before each experiment. Cells maintained at 37 °C served as the control (N) group. After treatment, cells were collected for total RNA extraction and qRT-PCR analysis, and culture medium was collected for glucose and lactate measurements. All cell experiments were performed in triplicate (three independent biological replicates).

#### 2.6.1. RNA Extraction and RT-qPCR

Total RNA was extracted from cultured DF-1 cells using TRIzol reagent (Invitrogen, Carlsbad, CA, USA). cDNA was synthesized using the PrimeScript^®^ 1st Strand cDNA Synthesis Kit (Takara Bio Inc., Shiga, Japan). Real-time PCR was performed using Bestar™ Real-time PCR Master Mix (DBI Bioscience, Dresden, Germany) on a CFX96 system (Bio-Rad Laboratories, Hercules, CA, USA). Each 20 µL reaction contained 10 µL SYBR Premix Ex Taq™ (Takara Bio Inc., Shiga, Japan), 0.2 µL of each primer (10 µM), and 1 µL cDNA. PCR conditions were: 95 °C for 2 min, followed by 40 cycles of 95 °C for 15 s and 60 °C for 20 s (signal collection), with a final melt curve from 65 to 95 °C (0.5 °C increments). β-actin was used as the internal reference, and relative expression was calculated using the 2^−ΔΔCT^ method. Primer specificity was validated by melt curve analysis (single peak) and by sequencing of PCR products. Primer amplification efficiency was determined from standard curves (serially diluted cDNA) and ranged from 95% to 105% for all primer pairs. Primer sequences are listed in Table 1.

#### 2.6.2. Vector Construction and Cell Transfection

siRNAs targeting chicken *ALDOB* and *HSP90B1* were chemically synthesized by Wuhan GeneCreate Biological Engineering Co., Ltd. (Wuhan, China) (Table 2). For overexpression, the complete coding sequences of *ALDOB* and *HSP90B1* were cloned into the pcDNA3.1 vector to generate pcDNA3.1-c*ALDOB* and pcDNA3.1-c*HSP90B1*, respectively; empty pcDNA3.1 was used as the negative control. DF-1 cells were seeded in 6-well plates at a density of 1–2 × 10^5^ cells/well and transfected with the respective constructs using Lipofectamine 3000 (Invitrogen, Carlsbad, CA, USA) according to the manufacturer’s instructions. Transfection efficiency was assessed by parallel transfection of a GFP-expressing plasmid (pcDNA3.1-EGFP) under identical conditions; efficiency was consistently >70% as determined by flow cytometry. Knockdown efficiency was validated by RT-qPCR, showing >75% reduction in target gene mRNA levels at 48 h post-transfection.

#### 2.6.3. Glucose and Lactic Acid Determination

Glucose concentration in the culture medium was quantified using a Glucose Detection Kit (Nanjing Jiancheng Bioengineering Institute, Nanjing, China). Glucose consumption was calculated as:

Glucose consumption (mmol/10^6^ cells) = (C_initial − C_final)/cell number

where C_initial is the glucose content in the original medium, and C_final is the glucose content in the post-transfection medium after 48 h. Lactic acid content was measured using a Lactic Acid Test Kit (Nanjing Jiancheng Bioengineering Institute). Lactic acid production was calculated as:

Lactic acid production (mmol/10^6^ cells) = (L_final − L_initial)/cell number

where L_initial is the lactic acid content in the original medium, and L_final is the lactic acid content in the post-treatment medium after 48 h.

### 2.7. Statistical Analysis

Data were processed using Microsoft Excel 2013 and analyzed with R software (version 4.2.1, R Core Team, Vienna, Austria, 2022). Before parametric tests, assumptions of normality (Shapiro–Wilk test, *p* > 0.05) and homogeneity of variances (Levene’s test, *p* > 0.05) were verified. When assumptions were violated, data were log-transformed, or non-parametric tests (Mann–Whitney U test) were used. Differences between the two groups were evaluated by an independent samples *t*-test (or Mann–Whitney U test). Multiple comparisons were performed by one-way ANOVA followed by Tukey’s honest significant difference (HSD) post hoc test. To account for multiple testing, a false discovery rate (FDR) correction was applied when appropriate, and adjusted *p*-values are reported. Effect sizes (Cohen’s *d* for *t*-tests; partial *η*^2^ for ANOVA) and 95% confidence intervals for mean differences are provided in the Supplementary Materials. Data are expressed as mean ± standard error (SE). Statistical significance was set at *p* < 0.05. The term “extremely significant” or “significant” was not used; instead, exact *p*-values are reported, with *p* < 0.05 considered significant and *p* < 0.01 considered highly significant but described as “*p* < 0.01” without superlatives.

All sample sizes (*n*) are indicated in the respective figure legends and tables. In animal experiments, *n* refers to the number of biological replicates (individual birds), with 6 birds per group per time point for meat quality and flavor analyses, unless stated otherwise. For cell experiments, *n* refers to the number of independent biological replicates (three per treatment).

## 3. Results

### 3.1. Effects of Chronic Heat Stress on Growth Performance and Flavor Substance Deposition

**Figure 1:**
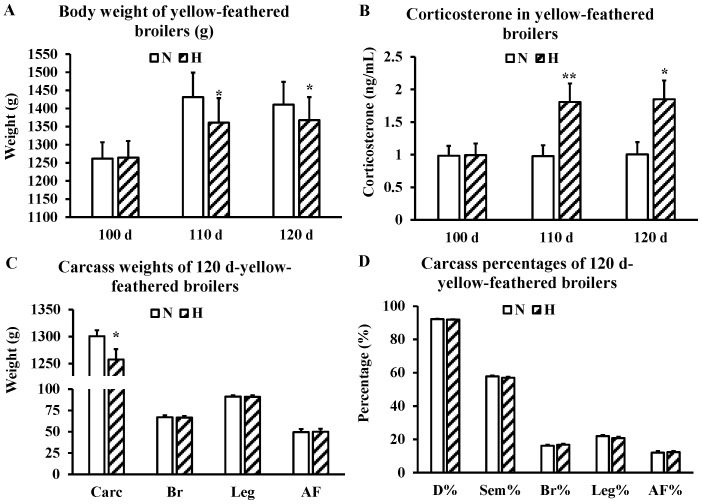
Growth performance and slaughter traits of slow-growing yellow-feathered broilers. (**A**) Body weight at 100, 110, and 120 days of age; (**B**) Plasma corticosterone concentrations in yellow-feathered broilers; (**C**,**D**) Carcass traits at 120 days of age: carcass weight (Carc), breast muscle weight (Br), leg muscle weight (Leg), abdominal fat weight (AF), dressing percentage (D%), semi-eviscerated percentage (Sem%), breast muscle percentage (Br%), leg muscle percentage (Leg%), and abdominal fat percentage (AF%). Abbreviations: N, non-heat stress group; H, heat stress group. *n* = 30 per group. Data are expressed as mean ± SD. * *p* < 0.05; ** *p* < 0.01.

Chronic heat stress significantly impaired the weight gain of yellow-feathered broilers compared with the N group. The growth experiment started at 100 d, and body weight differences between groups were statistically significant at both 110 d (*p* < 0.01) and 120 d (*p* < 0.05) (Figure 1A). Furthermore, chronic heat stress significantly increased plasma corticosterone levels, with the H group showing 1.8- and 1.9-fold higher concentrations at 110 and 120 days of age, respectively, compared to the N group (Figure 1B). Assessment of slaughter performance at 120 d revealed a significant reduction in carcass weight in the H group, while other slaughter traits showed no significant change (Figure 1C,D).

Regarding free amino acid content in muscle tissue (Table 3), heat stress significantly (*p* < 0.01) increased the concentrations of β-alanine and ornithine in both breast and leg muscles. In the leg muscles, phosphoserine and β-aminoisobutyric acid were significantly reduced (*p* < 0.01), and tyrosine was significantly decreased (*p* <0.05). No significant differences in total free amino acid content were observed between groups in either muscle type. With respect to fatty acid composition (Table 4), heat stress significantly elevated arachidic acid (C20:0) content in the breast muscle (*p* < 0.05), whereas no significant fatty acid differences were detected in leg muscles.

### 3.2. Changes in Meat Quality Traits During Post-Slaughter Cold Storage

**Figure 2:**
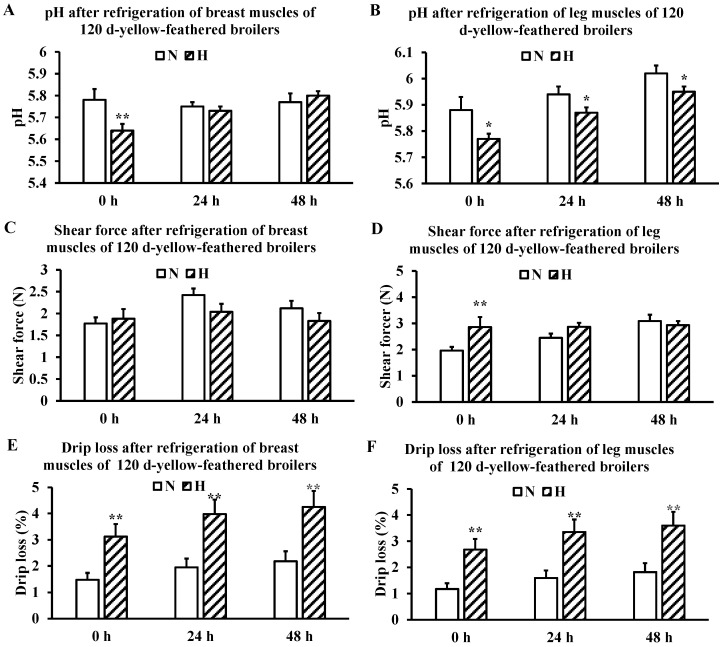
pH, shear force, and drip loss of breast and leg muscles of yellow-feathered broilers at 120 days of age following 0, 24, and 48 h of refrigeration at 4 °C. (**A**) Breast muscle pH; (**B**) leg muscle pH; (**C**) breast muscle shear force; (**D**) leg muscle shear force; (**E**) breast muscle drip loss; (**F**) leg muscle drip loss. *n* = 6 per group. Data are expressed as mean ± SD. * *p* < 0.05; ** *p* < 0.01.

Breast muscle pH was significantly reduced in the H group at 0 h but did not differ significantly from the N group following refrigeration (Figure 2A). Leg muscle pH in the H group was significantly lower than that in the N group at all refrigeration timepoints (0, 24, and 48 h; *p* < 0.05), indicating a more sustained acidification effect of heat stress on leg muscles (Figure 2B). No significant change in breast muscle shear force was observed after heat stress (Figure 2C). In contrast, leg muscle shear force was significantly elevated in the H group at 0 h post-slaughter (*p* < 0.01), with no significant difference compared with the N group after refrigeration (Figure 2D). Chronic heat stress significantly increased drip loss in both breast (Figure 2E) and leg muscles (Figure 2F) at each refrigeration time point (0, 24, and 48 h), with the H group consistently showing higher drip loss values than the N group, indicating that heat stress reduces muscle water-holding capacity.

During cold storage, leg muscle pH in both groups showed a gradual upward trend (Figure 2B). In the N group, the shear force of breast muscles increased from 0 h to 24 h and then decreased slightly by 48 h, while the leg muscle shear force increased progressively over the 48 h storage period (Figure 2C,D). Drip loss in both groups gradually increased with prolonged refrigeration (Figure 2E,F).

Meat color indices of breast muscles (L*, a*, b*) showed no significant differences between groups across all refrigeration timepoints (Table 5). For leg muscles (Table 6), L* and b* were significantly increased while a* was significantly decreased in the H group at 0 h (*p* < 0.01). The elevated L* in the H group persisted significantly throughout the refrigeration period (*p* < 0.05 at 24 h; *p* < 0.01 at 48 h). These findings collectively indicate that heat stress exerted a markedly greater negative effect on leg muscle meat color than on breast muscle, and likely promotes whitening, yellowing, and reduced redness—all characteristics consistent with PSE-like meat formation.

### 3.3. Effects of Heat Stress on Cellular Acid Production and Tissue Gene Expression

**Figure 3:**
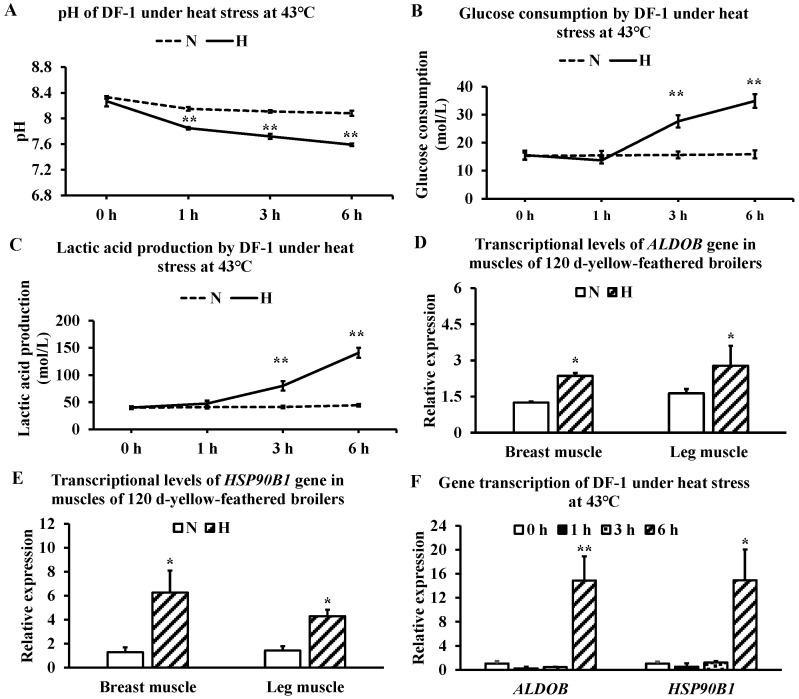
Effects of heat stress on cellular acid production and tissue gene expression. (**A**) pH changes in DF-1 culture medium under 43 °C heat stress; (**B**) glucose consumption by DF-1 cells under 43 °C heat stress; (**C**) lactic acid production by DF-1 cells under 43 °C heat stress; (**D**) *ALDOB* gene expression in tissues of slow-growing yellow-feathered broilers at 120 days; (**E**) *HSP90B1* gene expression in tissues of slow-growing yellow-feathered broilers at 120 days; (**F**) *ALDOB* and *HSP90B1* gene expression in DF-1 cells under heat stress at 43 °C. Abbreviations: DF-1, chicken embryo fibroblast cell line; *ALDOB*, aldolase B; *HSP90B1*, heat shock protein 90 beta family member 1 (also known as *GRP94*). (**A**–**C**) Data are expressed as mean ± SD; (**D**–**F**) Data are expressed as mean ± SE. * *p* < 0.05; ** *p* < 0.01.

Heat stress at 43 °C induced a progressive and highly significant decrease in DF-1 culture medium pH (Figure 3A), accompanied by significant increases in glucose consumption (Figure 3B) and lactic acid production (Figure 3C). Following 21 days of chronic heat stress in vivo, *ALDOB* expression in breast and leg muscles was significantly elevated approximately 1-fold above control levels (Figure 3D). *HSP90B1* expression in breast and leg muscles was significantly upregulated by approximately 5-fold and 3-fold, respectively (Figure 3E). In the DF-1 cell model, both *ALDOB* and *HSP90B1* transcriptional levels were significantly increased approximately 14-fold after 6 h of heat stress at 43 °C (Figure 3F).

### 3.4. Effects of ALDOB and HSP90B1 on Glycometabolism at the Cellular Level

**Figure 4:**
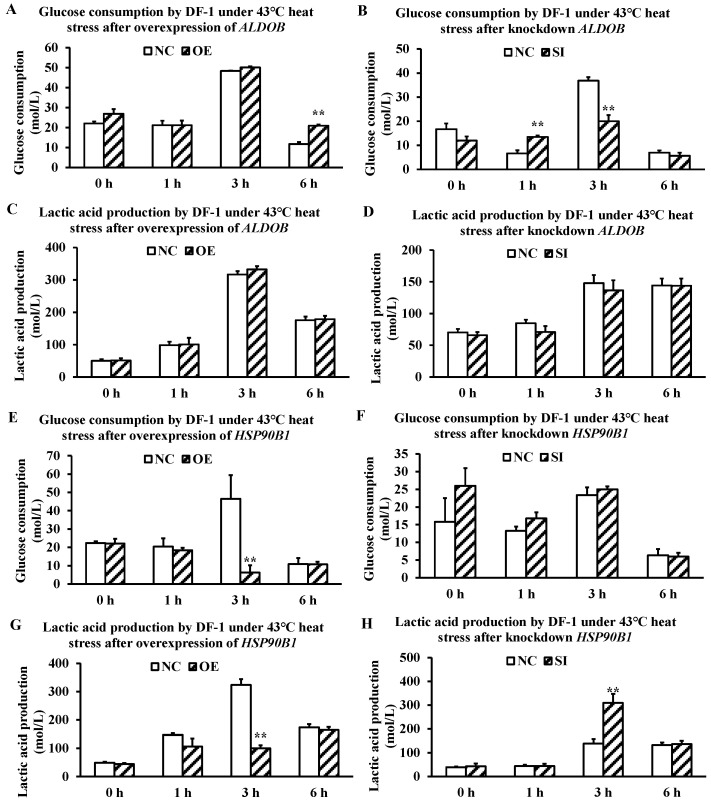
Effects of *ALDOB* and *HSP90B1* gene expression on glycometabolism in DF-1 cells under 43 °C heat stress. (**A**) Glucose consumption after *ALDOB* overexpression; (**B**) glucose consumption after *ALDOB* knockdown; (**C**) lactic acid production after *ALDOB* overexpression; (**D**) lactic acid production after *ALDOB* knockdown; (**E**) glucose consumption after *HSP90B1* overexpression; (**F**) glucose consumption after *HSP90B1* knockdown; (**G**) lactic acid production after *HSP90B1* overexpression; (**H**) lactic acid production after *HSP90B1* knockdown. Abbreviations: NC, negative control (empty vector or scrambled siRNA); OE, overexpression; SI, siRNA-mediated knockdown; DF-1, chicken embryo fibroblast cell line; *ALDOB*, aldolase B; *HSP90B1*, heat shock protein 90 beta family member 1 (*GRP94*). Data are expressed as mean ± SD. ** *p* < 0.01.

Overexpression of *ALDOB* in DF-1 cells resulted in a significant (~10-fold) increase in glucose consumption after 6 h of heat stress at 43 °C (Figure 4A). Conversely, siRNA-mediated knockdown of *ALDOB* caused a significant (~5-fold) increase in glucose consumption at 1 h, followed by a significant (~15-fold) decrease at 3 h, returning to baseline levels at 6 h (Figure 4B). Neither overexpression nor knockdown of *ALDOB* had a significant effect on lactic acid production (Figure 4C,D).

Overexpression of *HSP90B1* in DF-1 cells led to a significant (~40-fold) reduction in glucose consumption after 3 h of heat stress at 43 °C (Figure 4E), while knockdown of *HSP90B1* had no significant effect on glucose consumption (Figure 4F). Regarding lactic acid production, overexpression of *HSP90B1* significantly suppressed lactic acid output (~200-fold reduction, *p* < 0.01) after 3 h of heat stress (Figure 4G), whereas knockdown of *HSP90B1* caused a significant (~150-fold) increase in lactic acid production (Figure 4H). Together, these results demonstrate that *ALDOB* promotes cellular glucose consumption under heat stress, whereas *HSP90B1* functions as a suppressor of lactic acid production during heat-stress-induced glycometabolic responses.

## 4. Discussion

### 4.1. Effects of Heat Stress on Growth Performance

Chronic heat stress significantly reduced body weight at both 110 and 120 d (*p* < 0.05), consistent with previous reports that heat-exposed broilers exhibit lower feed intake and increased catabolism of endogenous fat and protein to meet thermoregulatory demands [2]. The reduction in carcass weight at 120 d further supports the negative impact of sustained cyclic heat stress on growth performance in slow-growing yellow-feathered broilers.

### 4.2. Effects of Heat Stress on Flavor-Related Compounds

Flavor precursors in meat include free amino acids and fatty acids, which contribute to taste and aroma through Maillard reactions and lipid degradation during cooking [26]. In the present study, chronic heat stress significantly increased β-alanine and ornithine in both breast and leg muscles, while reducing tyrosine, phosphoserine, and β-aminoisobutyric acid in leg muscles. These changes in the amino acid profile may influence the potential sensory properties of the meat; however, without direct sensory analysis, their actual impact on taste perception (e.g., sourness or bitterness) cannot be conclusively determined. β-Aminoisobutyric acid (BAIBA) is an exercise-induced cytokine that promotes adipose tissue browning [15]; its reduction under heat stress suggests possible impairment of thermogenic adaptation. Regarding fatty acids, the significant elevation of arachidic acid (C20:0) in breast muscle may reflect increased saturated fatty acid synthesis as a cellular homeostatic response. Nevertheless, the nutritional and sensory consequences of this change remain speculative and require further investigation.

### 4.3. Effects of Heat Stress on Meat Quality During Cold Storage

Muscle pH is determined by multiple factors, including H^+^ release from ATP hydrolysis, lactic acid accumulation from anaerobic glycolysis, creatine phosphate breakdown, and the intrinsic buffering capacity of muscle proteins [27]. Notably, postmortem pH decline is primarily driven by the hydrolysis of ATP to ADP and inorganic phosphate, which releases H^+^ ions; lactic acid accumulation contributes to but does not solely determine the extent of pH reduction [17,28]. Although heat stress significantly lowered pH in both breast and leg muscles at 0 h, the persistent acidification in leg muscles throughout 48 h of refrigeration indicates a more sustained effect on this muscle type. Regarding the gradual increase in muscle pH during cold storage, it should be noted that this phenomenon is not anomalous but rather a common feature of postmortem meat storage. In the early postmortem period (0–24 h), pH rapidly declines to its lowest point due to H^+^ accumulation from ATP hydrolysis and glycolysis [29]. As refrigeration extends to 48 h and beyond, pH slowly increases, driven by two main mechanisms. First, endogenous proteases (e.g., calpains, cathepsins) are activated and degrade myofibrillar and sarcoplasmic proteins, releasing weakly alkaline peptides and free amino acids that neutralize some H^+^. Second, even under refrigerated conditions (4 °C), psychrotrophic microorganisms (e.g., *Pseudomonas* spp., Enterobacteriaceae) can slowly proliferate and metabolize amino acids to produce ammonia (NH_3_) and biogenic amines (e.g., putrescine, cadaverine). These alkaline substances accept H^+^ to form ammonium ions (NH_4_^+^), thereby consuming free H^+^ and raising pH [17,29,30,31]. The gradual pH increase observed in leg muscles after 24 h in the present study is fully consistent with this well-established description and does not contradict fundamental meat science theories.

Pre-slaughter heat stress immediately increased leg muscle shear force at 0 h post-slaughter (H group vs. N group, *p* < 0.01), indicating accelerated rigor onset. This is consistent with the known effect of antemortem stress on postmortem energy metabolism, where rapid ATP depletion promotes irreversible actin-myosin cross-bridge formation, leading to increased toughness [32,33]. Regarding the changes in shear force during cold storage, an unexpected observation was the progressive increase in leg muscle shear force of the N group (from 1.96 N at 0 h to 3.09 N at 48 h), while shear force of the H group remained relatively stable after 24 h (Figure 2D). In normal postmortem aging, shear force typically decreases due to proteolytic degradation of myofibrillar proteins. The increase observed in the N group may be attributable to several factors. First, the 48 h aging period at 4 °C might be insufficient to induce detectable tenderization in leg muscles of this slow-growing yellow-feathered breed, which may have lower calpain activity or higher collagen content compared to breast muscle. Second, the rapid cooling rate (from 38 °C to 4 °C within 90 min) could have caused cold shortening in leg muscles, leading to sarcomere shortening and increased resistance to shearing [32]. Third, the lack of a statistically significant increase (as indicated by overlapping error bars in Figure 2D) suggests that the numerical trend may be within biological variation; further studies with extended aging (e.g., 7 days) and direct sarcomere length measurement are needed. In contrast, breast muscle shear force in both groups peaked at 24 h and declined by 48 h (Figure 2C), which is closer to the expected pattern of rigor development followed by partial tenderization. The lack of a clear tenderization in leg muscles within 48 h warrants further investigation into muscle-type-specific proteolytic systems.

Heat stress caused marked changes in leg meat color: increased L* and b* and decreased a* at 0 h, with elevated L* persisting for 48 h. These characteristics (paler, more yellow, less red) are typical of PSE-like meat [4,34,35]. The lesser effect on breast meat color may reflect differences in muscle fiber type composition and metabolic rate between breast and leg muscles, a possibility that warrants further investigation.

### 4.4. Roles of ALDOB and HSP90B1 in Glycometabolism Under Heat Stress

Chronic heat stress upregulated both *ALDOB* and *HSP90B1* in breast and leg muscles, as well as in heat-exposed DF-1 cells. These findings establish a consistent link between heat stress and altered expression of glycometabolism-related genes.

Overexpression of *ALDOB* significantly increased glucose consumption in heat-stressed DF-1 cells, whereas knockdown produced opposite dynamics. These results confirm that *ALDOB* participates in the early glycolytic response to heat stress. However, neither overexpression nor knockdown of *ALDOB* significantly affected lactic acid production. Therefore, *ALDOB* may influence upstream steps of glycolysis (glucose uptake and phosphorylation flux) without directly modulating terminal lactate generation. Consequently, its contribution to post-mortem muscle acidification, if any, is likely indirect and requires further investigation.

*HSP90B1* overexpression markedly suppressed lactic acid production in heat-stressed DF-1 cells, whereas knockdown increased lactate output. These observations suggest that *HSP90B1* functions as a negative regulator of lactic acid accumulation under heat stress. One plausible but unconfirmed mechanism involves *HSP90B1* interaction with glucocorticoid receptors: glucocorticoids, whose secretion is elevated during stress, bind to cytoplasmic receptors complexed with HSP90, leading to nuclear translocation and enhanced transcription of gluconeogenic genes [36,37]. Enhanced gluconeogenesis would reduce net glycolytic flux and lactate production. However, this interpretation remains hypothetical. Direct evidence—such as glucocorticoid receptor antagonists, reporter assays, or pathway-specific inhibitors—is required to validate the proposed mechanism. Future studies should also examine whether *HSP90B1* affects lactate transport or degradation via other pathways.

### 4.5. Study Limitations

Several limitations should be acknowledged. First, the sample size for flavor analysis (*n* = 6 per group) is small, and no sensory evaluation was performed; therefore, conclusions about taste perception are preliminary. Second, we did not measure physiological stress indicators (e.g., plasma corticosterone, respiration rate, body temperature), which limits the ability to confirm the biological relevance of the heat stress model. Third, water-holding capacity—a critical parameter for PSE meat classification—was not assessed. Fourth, the proposed glucocorticoid-mediated mechanism for *HSP90B1* was not directly tested; it remains a hypothesis requiring experimental validation. Fifth, the study focused on one slow-growing yellow-feathered breed; generalizability to fast-growing commercial broilers may be limited. Finally, the use of an outdated software version (SPSS 11.5) in the original analysis has been addressed by re-analysis using R (version 4.2.1); all reported results are consistent with the original findings.

## 5. Conclusions

Chronic cyclic heat stress impaired growth performance, altered flavor-related amino acid and fatty acid profiles, and promoted PSE-like meat deterioration—particularly in leg muscles—of slow-growing yellow-feathered broilers. Post-mortem pH decline is primarily driven by ATP hydrolysis, with lactic acid accumulation playing a secondary modulatory role. The unexpected changes in shear force during refrigeration may involve cold shortening and muscle-type differences, requiring further study. Heat stress upregulated *ALDOB* and *HSP90B1*; functional assays showed that *ALDOB* enhances glucose consumption, whereas *HSP90B1* suppresses lactic acid production. These findings offer a basis for understanding glycometabolic regulation under heat stress, although the proposed mechanisms and the lack of sensory analysis warrant additional validation.

## Figures and Tables

**Table 1:** Primers used for RT-qPCR of target genes and the internal reference gene.

Primer Name	Primer Sequence (5′–3′)
β-actin-F	TCATTGTGCTAGGTGCCA
β-actin-R	CCTCTTCCAGCCATCTTT
*ALDOB*-F	TGGTGGTGGGAATAAAGC
*ALDOB*-R	GAGGGCGTTGTGCTACTG
*HSP90B1*-F	ATGACATAAAACCAATCT
*HSP90B1*-R	AGCAGTAAAATGGATGTA

**Table 2:** siRNA sequence information for c*ALDOB* and c*HSP90B1* interference fragments.

Duplex Name	Strand	siRNA Sequence (5′–3′)
*ALDOB*	Sense	GCAGGAACAAAUGGAGAAATT
	Antisense	UUUCUCCAUUUGUUCCUGCTT
*HSP90B1*	Sense	GCACAACAAUGAUACCCAATT
	Antisense	UUGGGUAUCAUUGUUGUGCTT
siRNA-NC	Sense	UUCUCCGAACGUGUCACGUTT
	Antisense	ACGUGACACGUUCGGAGAATT

**Table 3:** Free amino acid content (mg/100 g wet weight) of breast and leg muscles of slow-growing yellow-feathered broilers at 120 days of age.

FAA (mg/100 g)	Breast Muscles			Leg Muscles		
	N Group	H Group	*p*-Value	N Group	H Group	*p*-Value
Phosphoserine	9.13 ± 0.10	9.47 ± 0.28	0.2245	17.74 ± 1.84	6.84 ± 0.14	0.0010 **
Taurine	22.67 ± 2.43	26.55 ± 1.44	0.2689	503.7 ± 35.61	467.30 ± 42.55	0.5365
Aspartic acid	3.21 ± 0.68	2.86 ± 1.28	0.8010	30.09 ± 10.75	8.04 ± 2.86	0.0947
Threonine	23.40 ± 2.14	18.72 ± 2.76	0.2307	52.00 ± 12.12	30.30 ± 3.41	0.1357
Serine	28.65 ± 0.87	27.78 ± 5.49	0.8604	69.73 ± 14.09	55.30 ± 8.81	0.4186
Valine	24.03 ± 1.11	23.43 ± 5.25	0.9014	38.82 ± 7.26	23.71 ± 3.41	0.1087
Methionine	15.68 ± 1.42	16.71 ± 4.10	0.7982	24.13 ± 4.48	13.76 ± 2.91	0.1001
Isoleucine	21.95 ± 1.83	17.92 ± 5.50	0.4643	31.72 ± 6.16	18.58 ± 3.53	0.1137
Leucine	42.19 ± 3.05	36.81 ± 12.34	0.6439	63.81 ± 13.37	36.06 ± 8.58	0.1314
Tyrosine	19.25 ± 0.82	18.42 ± 3.15	0.7802	24.07 ± 3.84	13.09 ± 2.04	0.0449 *
Asparagine	8.22 ± 0.32	6.92 ± 1.87	0.4552	5.05 ± 3.13	7.08 ± 2.33	0.6226
Glutamic acid	44.08 ± 1.96	38.88 ± 9.05	0.5406	94.82 ± 14.92	67.64 ± 12.64	0.2139
Glutamine	9.60 ± 3.33	14.80 ± 4.14	0.3678	76.77 ± 4.18	75.89 ± 15.06	0.957
Glycine	17.69 ± 0.65	20.33 ± 4.88	0.5531	55.51 ± 5.64	46.88 ± 4.08	0.2608
Alanine	62.74 ± 4.50	81.10 ± 16.78	0.2758	149.80 ± 24.08	123.60 ± 10.61	0.3569
Phenylalanine	20.69 ± 1.41	20.15 ± 6.10	0.9244	36.83 ± 9.36	19.03 ± 4.47	0.1372
β-Alanine	2.72 ± 0.47	7.12 ± 0.88	0.005 **	10.03 ± 1.47	16.43 ± 1.23	0.0157 *
β-Aminoisobutyric acid	0.48 ± 0.17	0.33 ± 0.18	0.5740	1.34 ± 0.05	0.60 ± 0.08	0.0002 **
γ-Aminobutyric acid	0.26 ± 0.03	0.42 ± 0.09	0.0978	0.43 ± 0.10	0.79 ± 0.13	0.066
Histidine	19.07 ± 1.24	15.98 ± 4.53	0.4807	28.94 ± 5.64	16.13 ± 2.84	0.0887
3-Methylhistidine	0.60 ± 0.04	0.61 ± 0.16	0.9199	0.73 ± 0.15	0.60 ± 0.06	0.4552
1-Methylhistidine	1.14 ± 0.03	1.05 ± 0.19	0.5914	1.15 ± 0.07	0.90 ± 0.08	0.0635
Carnosine	892.50 ± 73.51	943.60 ± 30.77	0.5977	273.30 ± 49.40	351.50 ± 43.41	0.2795
Tryptophan	1389.60 ± 63.49	1424.20 ± 64.40	0.7239	398.5 ± 8.57	428.40 ± 24.72	0.2975
Ornithine	0.30 ± 0.03	1.10 ± 0.18	0.0031 **	1.30 ± 0.08	3.37 ± 0.68	0.0231 *
Lysine	37.52 ± 3.34	36.33 ± 10.71	0.9083	83.51 ± 18.81	38.03 ± 2.08	0.0531
Arginine	27.48 ± 4.77	23.50 ± 4.58	0.5849	63.67 ± 12.26	35.51 ± 3.66	0.07
Total amino acids	2729.00 ± 90.47	2883.60 ± 95.89	0.2999	2155.00 ± 12.80	1874.4 ± 54.05	0.1722

*n* = 6 per group. Data are expressed as mean ± SE. * *p* < 0.05; ** *p* < 0.01. N group: non-heat stress group; H group: heat stress group. FAA: Free amino acid.

**Table 4:** Fatty acid composition (% of total fatty acids) of breast and leg muscles of slow-growing yellow-feathered broilers at 120 days of age.

Fatty Acid (%)	Breast Muscles			Leg Muscles		
	N Group	H Group	*p*-Value	N Group	H Group	*p*-Value
C17:0	0.57 ± 0.08	0.42 ± 0.08	0.2131	0.20 ± 0.04	0.25 ± 0.06	0.4732
C17:1	0.35 ± 0.06	0.24 ± 0.04	0.1606	0.20 ± 0.00	0.20 ± 0.00	1
C18:0	7.48 ± 0.41	6.97 ± 0.38	0.3728	6.55 ± 0.83	6.48 ± 0.28	0.8829
C18:1cis	33.33 ± 1.25	34.90 ± 0.86	0.3261	36.73 ± 0.91	36.50 ± 0.71	0.8446
C18:2cis	19.97 ± 0.60	21.02 ± 1.01	0.3931	21.73 ± 0.95	21.97 ± 1.20	0.8816
C18:3cis	0.67 ± 0.03	0.68 ± 0.05	0.7805	0.75 ± 0.03	0.73 ± 0.03	0.7342
C12:0	0.28 ± 0.03	0.30 ± 0.02	0.6867	0.36 ± 0.02	0.33 ± 0.03	0.4178
C14:0	0.85 ± 0.04	0.88 ± 0.04	0.5826	0.92 ± 0.02	0.90 ± 0.04	0.6867
C14:1	0.15 ± 0.02	0.15 ± 0.02	1	0.20 ± 0.03	0.15 ± 0.02	0.1739
C15:0	2.03 ± 0.37	1.57 ± 0.36	0.3899	0.55 ± 0.11	0.77 ± 0.17	0.3131
C16:0	24.33 ± 0.50	23.83 ± 0.47	0.482	23.80 ± 0.50	23.35 ± 0.57	0.5670
C16:1	3.88 ± 0.41	4.10 ± 0.45	0.7283	4.95 ± 0.37	4.93 ± 0.53	0.9799
C20:0	0.12 ± 0.02	0.18 ± 0.02	0.0364 *	0.20 ± 0.03	0.16 ± 0.02	0.2967
C20:1	0.25 ± 0.02	0.27 ± 0.02	0.5995	0.30 ± 0.00	0.27 ± 0.02	0.1449
C20:2	0.24 ± 0.02	0.25 ± 0.02	0.7699	0.23 ± 0.02	0.18 ± 0.02	0.1039
C22:0	0.32 ± 0.06	0.40 ± 0.04	0.292	0.23 ± 0.02	0.22 ± 0.02	0.5490
C20:4	2.87 ± 0.62	3.62 ± 0.64	0.417	1.60 ± 0.26	1.90 ± 0.30	0.4650
C24:1	0.62 ± 0.13	0.85 ± 0.17	0.2994	0.43 ± 0.08	0.45 ± 0.08	0.8918
C22:5	0.25 ± 0.06	0.26 ± 0.06	0.9062	0.15 ± 0.03	0.12 ± 0.02	0.4071
C22:6	0.47 ± 0.08	0.46 ± 0.08	0.9536	0.17 ± 0.02	0.20 ± 0.03	0.3893

*n* = 6 per group. Data are expressed as mean ± SE. * *p* < 0.05. N group: non-heat stress group; H group: heat stress group.

**Table 5:** Meat color data (ΔL*, Δa*, Δb*) after refrigeration of breast muscles of slow-growing yellow-feathered broilers at 120 days of age.

Color Index	0 h			24 h			48 h		
	N	H	*p*-Value	N	H	*p*-Value	N	H	*p*-Value
ΔL*	61.74 ± 1.06	63.67 ± 1.20	0.172	61.12 ± 0.85	62.78 ± 0.85	0.238	61.08 ± 0.72	62.69 ± 1.15	0.2520
Δa*	7.90 ± 0.37	6.86 ± 0.47	0.079	8.45 ± 0.48	7.56 ± 0.31	0.131	8.10 ± 0.48	7.08 ± 0.32	0.0820
Δb*	9.29 ± 0.37	8.81 ± 0.38	0.351	8.86 ± 0.40	8.60 ± 0.34	0.62	9.64 ± 0.36	9.09 ± 0.31	0.2900

*n* = 6 per group. Data are expressed as mean ± SE. N group: non-heat-stress group; H group: heat-stress group.

**Table 6:** Meat color data (ΔL*, Δa*, Δb*) after refrigeration of leg muscles of slow-growing yellow-feathered broilers at 120 days of age.

Color Index	0 h			24 h			48 h		
	N	H	*p*-Value	N	H	*p*-Value	N	H	*p*-Value
ΔL*	58.98 ± 0.56	62.62 ± 1.21	0.006 **	59.55 ± 0.99	62.74 ± 1.03	0.016 *	58.29 ± 0.77	63.16 ± 0.84	0.0003 **
Δa*	12.71 ± 0.29	11.05 ± 0.33	0.003 **	11.01 ± 0.55	11.58 ± 0.43	0.296	11.88 ± 0.30	11.40 ± 0.33	0.3800
Δb*	6.73 ± 0.56	8.77 ± 0.48	0.004 **	7.45 ± 0.55	7.66 ± 0.42	0.757	8.30 ± 0.50	7.25 ± 0.21	0.1360

*n* = 6 per group. Data are expressed as mean ± SE. * *p* < 0.05; ** *p* < 0.01. N group: non-heat stress group; H group: heat stress group.

## Data Availability

The authors confirm that the data supporting the findings of this study are available within the article and its Supplementary Materials. Raw data supporting the conclusions of this article will be made available by the corresponding authors upon reasonable request.

## Supplementary Materials

The following supporting information can be downloaded at: https://www.mdpi.com/article/10.3390/metabo16050298/s1. Table S1: Raw data corresponding to Figure 1 (Growth performance and slaughter traits of slow-growing yellow-feathered broilers). Table S2: Raw data corresponding to Figure 2 (pH, shear force, and drip loss of breast and leg muscles at 0, 24, and 48 h of refrigeration at 4 °C). Table S3: Raw data corresponding to Figure 3 (Effects of heat stress on cellular acid production and tissue gene expression). Table S4: Raw data corresponding to Figure 4 (Effects of ALDOB and HSP90B1 gene expression on glycometabolism in DF-1 cells under 43 °C heat stress).
